# *Pulicaria incisa* (Lam.) DC. as a Potential Source of Antioxidant, Antibacterial, and Anti-Enzymatic Bioactive Molecules: Phytochemical Constituents, In Vitro and In Silico Pharmacological Analysis

**DOI:** 10.3390/molecules28217439

**Published:** 2023-11-05

**Authors:** Mousa Alreshidi, Mohammad A. Abdulhakeem, Riadh Badraoui, Giuseppe Amato, Lucia Caputo, Laura De Martino, Filomena Nazzaro, Florinda Fratianni, Carmen Formisano, Vincenzo De Feo, Mejdi Snoussi

**Affiliations:** 1 Department of Biology, College of Science, University of Ha’il, Hail 2440, Saudi Arabia; mo.alreshidi@uoh.edu.sa (M.A.); s20200103@uoh.edu.sa (M.A.A.); m.snoussi@uoh.edu.sa (M.S.); 2 Section of Histology-Cytology, Medicine Faculty of Tunis, University of Tunis El Manar, Tunis 1007, Tunisia; 3 Department of Pharmacy, University of Salerno, 84084 Fisciano, Italy; gamato@unisa.it (G.A.); ldemartino@unisa.it (L.D.M.); 4 Institute of Food Sciences, CNR, 83100 Avellino, Italyflorinda.fratianni@isa.cnr.it (F.F.); 5 Department of Pharmacy, School of Medicine and Surgery, University of Napoli Federico II, 80131 Napoli, Italy; caformis@unina.it; 6 Laboratory of Genetics, Biodiversity and Valorization of Bio-Resources (LR11ES41), Higher Institute of Biotechnology of Monastir, University of Monastir, Avenue Tahar Haddad, BP74, Monastir 5000, Tunisia

**Keywords:** *P. incisa* extract, phytochemical composition, antibacterial, antioxidant activities, anti-enzymatic activities, ADME, molecular docking

## Abstract

Plants with medicinal benefits are a crucial source of compounds for developing drugs. This study was designed to determine the chemical composition, antibacterial, antibiofilm, antioxidant, and anti-enzymatic activities of *Pulicaria incisa* (Lam.) DC. We also reported the molecular interaction between identified molecules and several receptors associated with antimicrobial and antibiofilm activities. A total of seventeen and thirteen compounds were identified in aqueous and methanolic extracts of *P. incisa*, respectively. The methanolic extract yielded a higher total content of polyphenols and flavonoids of about 84.80 ± 2.8 mg GAE/g and 28.30 ± 1.2 mg QE/g, respectively. Significant antibacterial activity was recorded for both extracts, with minimum inhibitory concentration (MIC) values ranging from 30 to 36 µg/mL, and the result was comparable to the reference antibiotic control. Antibiofilm assays revealed that both extracts were able to reduce the attachment of bacterial cells to 96-well plates, but the highest antibiofilm activity was recorded against *Staphylococcus aureus*. The methanolic extract also showed anti-enzymatic potency and high antioxidant activity, as demonstrated by all assays used, including DPPH, FRAP, and ABTS. These results were further validated by in silico approaches, particularly the molecular interaction of the identified compounds with the targeted receptors. These findings present *P. incisa* as a significant source of antibacterial, antibiofilm, antioxidant, and anti-enzymatic molecules.

## 1. Introduction

*Pulicaria* species (Asteraceae) are widely distributed in the Arabian region, including Saudi Arabia, as the plant survives in saline and arid environments [1,2]. The species have acclimatized to grow in these environments by inducing various morphological and biochemical alterations to combat undesirable conditions [3,4]. Some *Pulicaria* species are sometimes added to hot beverages, particularly tea, in some regions in the north of Saudi Arabia and have been comprehensively used in traditional medicine as an insect repellent and epilepsy remedy and for other economic purposes. They are habitually used as a medicine for influenza, gastrointestinal diseases, and joint inflammation [5]. The essential oil from *Pulicaria* species, particularly *P. undulata*, has been shown to have anticancer activities and substantial antioxidant properties [6].

*Pulicaria incisa* (Lam.) DC. has been characterized as a potential agent for treating heart diseases because it has a high quantity of unsaturated fatty acids able to reduce total triglyceride and cholesterol levels [7]. This species is characterized by its aromatic odor and essential oil content [6]. Indeed, *P. incisa* has been used as an ingredient of perfume, a tonic, a hypoglycemic, and an antispasmodic. It contains various biological molecules that may contribute to its ability to grow in arid environments; thus, it could be highly useful for antimicrobial activities due to the synthesis of the chemical constituents of arid plants. *P. incisa* was recently demonstrated to have efficient antioxidant activity, and based on this evidence, further evaluation of its potential uses as a drug agent for neurodegenerative and brain illnesses characterized by oxidative stress has been suggested [7,8]. The plant has yet to be comprehensively studied, but a few studies have reported its anti-inflammatory, antioxidant, and cytotoxic activities [9,10].

The exploration of bioactive molecules of plants with potential uses for treating human diseases, including diabetes, brain diseases, and microbial diseases, has been one of the most rigorous fields of scientific research. Therefore, natural products are increasingly used in the pharmaceutical industry as a foundation for bioactive molecules for the treatment of various human diseases. Furthermore, the investigation of herbs and plant-based products, as well as major pharmacological alternatives, remains essential. The biological activities, molecular docking, and dynamics of *P. incisa* have not yet been investigated, despite its assessed medicinal, biological, and environmental importance.

Therefore, this study aimed to characterize the chemical composition of *P. incisa* and subsequently identify potential bioactive molecules. This study also explored the antimicrobial, antibiofilm, antioxidants, and antidiabetic activities of aqueous and methanolic extracts of *P. incisa* using both in vitro and in silico approaches. The pharmacokinetic properties of the identified molecules in both the aqueous and methanolic extracts are described.

## 2. Results and Discussion

### 2.1. Chemical Analysis

**Figure 1:**
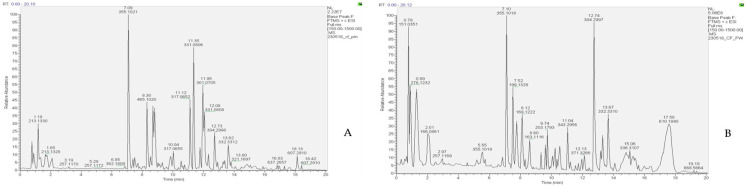
LC-MS profiles (base peak chromatogram) in positive ion mode of *Pulicaria incisa* (**A**) methanolic and (**B**) aqueous extracts.

LC-HRESIMS/MS analyses of the *P. incisa* extracts resulted in the separation and annotation of 17 components belonging to several classes of molecules (Table 1). Figure 1 reports a chromatogram of the two extracts. Compound **1** gave an [M + H]^+^ ion at *m*/*z* 294.1545, attributed to N-fructosyl (iso)leucine (C_12_H_23_O_7_N). The compound also yielded a major fragment at *m*/*z* 248.1492 (C_11_H_22_NO_5_), related to a loss of a carboxyl group, as reported by Pecio et al. [11], where the molecule was found in *Iphiona mucronata* (Forssk.) Asch. and Schweinf, a species belonging to the tribe Inuleae and chemotaxonomically close to *Inula* [11]. Another amino acid derivative was found in both the aqueous and methanolic extracts and identified as L-phenylalanine, a compound that yielded an [M + H]^+^ ion at *m*/*z* 166.0862 and has been frequently reported in the amino acid composition of different species of the *Pulicaria* genus [12,13]. Also in this case, the MS2 fragment at *m*/*z* 120.0806 was related to a loss of carboxyl group. Compound **3** gave an [M + H]^+^ ion at *m*/*z* 355.1019, which was attributed to an isomeric chlorogenic acid [9]. The analysis, based on the MS2 data, revealed a fragment at *m*/*z* 163.0391, related to the loss of quinic acid and also recently reported in the literature [14,15]. The compound was also found in other *Pulicaria* species [16].

The base peak at *m*/*z* 199.1328, observed in both chromatograms (Rt 7.52 mn), was attributed to the presence of (−)-hydroxy-dihydrobovolide. The compound showed an MS2 fragment at *m*/*z* 181.1222, corresponding to the loss of an OH group. The compound was previously found in the Asteraceae family [17]. Compounds **5** and **6** were two phenolic acids that gave base peaks at 339.0926 [M + H]^+^ and 369.1042 [M + H]^+^, respectively, corresponding to two protonated molecular forms of O-coumaroylquinic acid and feruloylquinic acid. The first acid gave an MS2 fragment with *m*/*z* 147.0438, related to a loss of quinic acid [18] (Yang et al., 2023); also, the second phenolic acid gave an MS2 fragment with *m*/*z* 177.0545, related to a loss of quinic acid. Both substances were previously identified in the same genus [18,19]. In addition, compound **7**, eluting at 8.12 mn in both extracts, showed a base peak at 169.1222 [M + H]^+^ and was identified as 8-hydroxycarvotanacetone, previously reported in a dichloromethane extract from *P. jaubertii* [20]. An MS/MS spectrum showed a fragmented ion peak at *m*/*z* 151.1108 which corresponded to the loss of a water molecule and at *m*/*z* 109.0658, produced by the loss of a C_3_H_6_ group.

The first flavonoid, compound **8**, eluted at 8.30 mn, gave a protonated molecular ion at *m*/*z* 465.1020, for which the molecular formula C_21_H_20_O_12_ was generated. The fragmentation of this compound, present only in the methanolic extract of *P. incisa*, was in agreement with isoquercetin, which gave an MS2 fragment at *m*/*z* 303.0496, corresponding to the protonated molecular ion of quercetin. Recently, a study reported the presence of this flavonoid in *P. incisa* [9].

The base peak at *m*/*z* 517.1329 [M + H]^+^, observed only in the methanolic extract (Rt 8.80), was attributed to the presence of an organic acid corresponding to (−)-3,5-dicaffeoylquinic acid. The fragmentation pathway revealed the presence of a peak at *m*/*z* 499.1226, corresponding to a loss of H_2_O, and a peak at *m*/*z* of 355.1019, corresponding to a protonated molecular ion of caffeoylquinic acid. Bakr and coworkers (2021) [9] reported the presence of this compound in the same species. Compound **10** gave an [M + H]^+^ ion at *m*/*z* 203.101792, attributed to calamenene, a sesquiterpene hydrocarbon usually found in plant essential oils. Moreover, the MS2 data analysis revealed a fragment at *m*/*z* 147.1165, corresponding to the losses of isopropyl and methyl groups. Recently, Askari et al. [21] reported the presence of the compound in the methanolic extract of *P. undulata*. The base peak at *m*/*z* 317.0652 [M + H]^+^, observed in both extracts (Rt 11.12), was attributed to the presence of rhamnetin. The fragment at *m*/*z* 299.0550 corresponded to the loss of H_2_O. The compound was previously found in the same species [19] with a similar fragmentation pattern. Compound **12** gave an [M + H]^+^ ion at *m*/*z* 331.0806, attributed to quercetin dimethyl ether. The fragment peaks observed in the MS spectra of the extracts showed the loss of a methyl group (15 amu), resulting in the fragmented ion at *m*/*z* 316.0573. Both quercetin 3,3′-dimethyl ether and quercetin 3,7-dimethyl ether were previously reported in *P. incisa* [22,23]. The base peak at *m*/*z* 287.0912 [M + H]^+^, observed only in the methanolic extract (Rt 11.77), was attributed to the presence of sakuranetin. The compound, previously reported in *Inula viscosa* L. [24], gave an MS2 fragment with *m*/*z* 167.0339, also reported in the literature [25], and the fragment corresponded to a loss of C_8_H_6_O [26].

Compound **14** gave an [M + H]^+^ ion at *m*/*z* 301.0705, corresponding to a protonated molecular form of chrysioerol, recently found in *P. incisa* by El-Sabagh and coworkers [19]. The compound produced a fragment with *m*/*z* 286.0784, related to the loss of a methyl group [27].

Compound **15** gave an [M + H]^+^ ion at *m*/*z* 315.0864, attributed to dihydroxy–dimethoxyflavone. In this case, the MS2 data analysis revealed a fragment at *m*/*z* 300.0733, corresponding to the loss of a methyl group. Several authors [28,29,30] have reported the presence of this compound among flavonoids found in the same genus. Compound **16** gave an [M + H]^+^ ion at *m*/*z* 345.0969, attributed to dihydroxy–trimethoxyflavone. The analysis revealed a fragment at *m*/*z* 330.0630, corresponding to the loss of the methyl group. The compound was reported among flavonoids found in methanolic extracts of the same species [19].

The base peak at *m*/*z* 451.1380, observed only in the methanolic extract (Rt 13.15), was attributed to the presence of 1-[9-(6-acetyl-5-hydroxy-2-benzofuranyl)-6,7,8,9-tetrahydro-2,6-dihydroxy-6-(hydroxymethyl)-3-dibenzofuranyl]-ethenone. The MS2 fragment with an *m*/*z* 402.3573 was consistent with the loss of H_2_O and CH_2_OH groups. The compound was previously reported in the Asteraceae family [31].

### 2.2. Antibacterial Activity

The minimum inhibitory concentration (MIC) was determined by selecting the lowest concentration of both aqueous and methanolic extracts that completely inhibited the growth of the tested pathogens in 96-well plates. The MICs of both extracts ranged from 30 to 36 µg/mL, whereas the MIC of the reference control antibiotic, tetracycline, ranged from 25 to 32 μg/mL (Table 2). The analysis revealed significant bacterial activities of both extracts against various Gram-positive (*Listeria monocytogenes*, *Staphylococcus aureus*) and Gram-negative pathogenic bacteria (*Acinetobacter baumannii*, *Escherichia coli*, *Pseudomonas aeruginosa*).

A similar finding was previously observed in *P. crispa* extracts [32]. In fact, the same authors reported that hexane fractions from *P. crispa* had antibacterial activities against four bacterial pathogens (*S. aureus*, *Klebsiella pneumoniae*, *E. coli*, and *P. aeruginosa*), with MIC values ranging from 62.5 to 125 μg/mL, and were able to affect influenza A virus at various stages of its lifecycle. However, another investigation revealed that *P. undulata* exhibited high activity against Gram-positive bacteria in comparison with Gram-negative bacteria with mean growth inhibition zones ranging from 11.5 ± 0.2 mm against methicillin-resistant *S. aureus* to 21.6 ± 0.1 mm for *Staphylococcus saprophyticus* ATCC 43867 [2].

Furthermore, *P. gnaphalodes* (Vent.) Boiss. has been shown to induce substantial antibacterial activity against *E. coli*, *Bacillus subtilis*, *S. aureus*, and *Pasteurella multocida* [33]. Interestingly, both fractions in the current study had identical MIC values of 30 ± 3 µg/mL and 35 ± 3 µg/mL when tested against *A. baumannii* and *Listeria monocytogenes*, respectively, and a similar MIC value was recorded for *P. aeruginosa*. The methanolic extract displayed the highest antibacterial activity against *E. coli*, with an MIC value of 32 ± 4 µg/mL.

Overall, the MIC values of both extracts exhibited similar potency against the corresponding bacteria and were comparable to the reference control antibiotic.

### 2.3. Antibiofilm Activity

The effects of the aqueous and methanolic extracts against mature biofilm were evaluated at the concentrations of 10 and 20 µg/mL, using the crystal violet biofilm test. The methanolic extract at 20 µg/mL induced the highest percentage of biofilm inhibition in *P. aeruginosa* (55.78%), followed by *S. aureus* and *E. coli* (51.2 and 44.62%, respectively) (Table 3). The extracts showed less activity against *L. monocytogenes* and *A. baumannii*. The inhibition correlated with an increase in their concentration. A recent publication indicated that *P. crispa* Sch. Bip. extracts induced various potencies on bacterial detachment from polystyrene surfaces [32]. It has been suggested that the interaction of the extracts is dependent on bacterial metabolism.

The antibiofilm activity of both extracts was also assessed using the 3-(4,5-dimethylthiazol-2-yl)-2,5-diphenyl-2H-tetrazolium bromide (MTT) assay (Table 4). This test was used to evaluate the effects of the two extracts, tested at the same concentrations on the metabolism of the cells included in the biofilm niches. Both the aqueous and methanolic extracts were unable to affect the metabolism of the cells of *A. baumannii*, *E. coli*, *L. monocytogenes*, and *P. aeruginosa* included in the biofilm, at both concentrations. Conversely, the extracts were significantly active in inhibiting the metabolism of *S. aureus*, and the highest inhibition was observed at the highest concentration of the aqueous extract (Table 4). Finally, the methanolic extract acted against the metabolism of *P. aeruginosa* at the highest concentration used. The obtained results confirmed the potential antibiofilm activity of species from the *Pulicaria* genus; in fact, a previous study demonstrated that *P. crispa* extract also had different degrees of antibiofilm activity, in that case, against some *K. pneumoniae* strains [34].

### 2.4. Anti-Enzymatic Activities

Enzyme inhibitors have great physiological and medical significance. In this study, the possible anti-enzymatic activities against acetylcholinesterase (AChE) and butyrylcholinesterase (BChE), key enzymes involved in the termination of fast cholinergic transmission [35], and α-amylase and α-glucosidase, involved in the development of diabetes mellitus (DM) [36], were evaluated. The methanolic extract was more active on cholinesterase than the aqueous extract, although the activity of both extracts was lower than that of galantamine used as a control (Table 5). Phenolic compounds can interact with amino acid residues in the active sites of AChE and BChE [35,37]. Quercetin has been reported as a strong inhibitor of both enzymes, which are involved in the pathology of Alzheimer’s disease [38]. Here, the activity of the *P. incisa* extracts could have been due to the presence of chlorogenic acid. Another study reported that this compound inhibited key enzymes in rat brains in vitro [39].

No previous studies reported on the activity of AChE or BChE for *P. incisa* extracts. Other species of *Pulicaria* genus were studied for their activity against these enzymes: Zardi-Bergaoui and coworkers analyzed the anti-AchE activity of five caryophyllene sesquiterpenes isolated from *P. vulgaris* ethanolic extract showing IC_50_ values ranging from 11.1 µg/mL to 55.3 µg/mL [40], and de la Luz Cádiz-Gurrea and coworkers studied the anti-enzymatic activities of *P. dysenterica* aqueous and methanolic extracts, and their results showed that methanolic extract was the most potent inhibitor of both cholinesterase [41]. Moreover, *P. diversifolia* and *P. stephanocarpa* chloroformic extract showed an AChE inhibition of 41.2% and 61.4%, respectively, at 200 µg/mL [42].

To that concern, our two extracts showed no activity against α-amylase with an IC_50_ value > 10 mg/mL with respect to acarbose, used as a positive control, which showed an IC_50_ of 11 µg/mL. Instead, against α-glucosidase, the *P. incisa* aqueous extract was more active than the *P. incisa* methanolic extract with an IC_50_ of 393.9 µg/mL, also lower than acarbose, which was used as a standard. This study reports for the first time the possible activity of *P. incisa* extracts against enzymes involved in the development of diabetes.

The role of phenolic compounds against α-amylase and α-glucosidase activity remains unclear [43], and in fact, even if flavonoids can inhibit starch digestion by inhibiting α-amylase activity [44], on the other hand, phenolic acids are poor inhibitors of these enzymes [45]. However, few studies described these possible activities for other species of *Pulicaria* genus, and in all cases, the major activity reported for different extracts was against α-glucosidase. A *P. dysenterica* water extract was found to be more active against α-glucosidase than methanolic extract, as analyzed by de la Luz Cádiz-Gurrea and coworkers [41]. *Pulicaria jaubertii* alcoholic and *n*-hexane extracts were also shown to be more active against α-glucosidase than against α-amylase [46].

### 2.5. Total Polyphenol Content (TPC) and Total Flavonoid Content (TFC)

The total content of polyphenols and flavonoids of the two extracts is reported in Table 6. The methanolic extract (84.80 ± 2.8 mg GAE/g) had a higher amount of TPC than the aqueous extract (56.6 ± 2.1 mg GAE/g). There is limited information on *P. incisa* in the literature. However, Mohamed and coworkers [47] reported that the polyphenol content of a methanolic extract of *P. incisa* was 61.22 mg GAE/g, which is higher than that of our study. The aqueous extract presented a lower amount of total flavonoid content than the methanolic extract (11.24 ± 0.8 and 28.30 ± 1.2 mg QE/g, respectively). These results are in agreement with the literature data [16].

### 2.6. Antioxidant Activity

The antioxidant activity of the extracts was evaluated through the DPPH and ABTS assays, their ability to neutralize radicals, and the FRAP assay. These tests allowed for the evaluation of their antioxidant activity targeting different reactive oxygen species. The DPPH assay allows for the quantification of substances that are involved through their reducing power, both in the transfer of electrons and hydrogen. The FRAP assay, by contrast, evaluates the substances that are exclusively involved in the transfer of electrons. Lastly, the ABTS assay assesses the antioxidant activity of both hydrophilic and lipophilic molecules in a wide pH range.

The DPPH test showed that both extracts had antioxidant activity, with IC_50_ values lower than the literature data, particularly for the methanolic extract [16]. In fact, the available literature reports the antioxidant activity of *P. crispa* and *P. petiolaris*, as evaluated by DPPH test [48,49].

The data obtained from the FRAP and ABTS assays confirmed the DPPH assay values. The methanolic extract had higher antioxidant activity in both tests. According to the FRAP results, the methanolic extract had a higher value of mmol (Fe^2+^)/g than the mmol (Fe^2+^)/g of the aqueous extract, and also in the ABTS assay, the aqueous extract showed lower activity than the methanolic extract (Table 6). No data are available in the literature about these two assays on *P. incisa*, although there is information regarding other *Pulicaria* species. Foudah and coworkers evaluated the antioxidant activity of a methanolic extract of *P. crispa* by DPPH and FRAP assays [50], whereas Kozarević and coworkers analyzed *P. dysenterica* (L.) Gaertn. [51]. These agree with those of this study, thus highlighting the antioxidant activity of some *P. incisa* species. Furthermore, the antioxidant activity shown by the two extracts was closely related to the presence of polyphenols and flavonoids [52].

### 2.7. Computational Analysis and Interaction Assay

**Figure 2:**
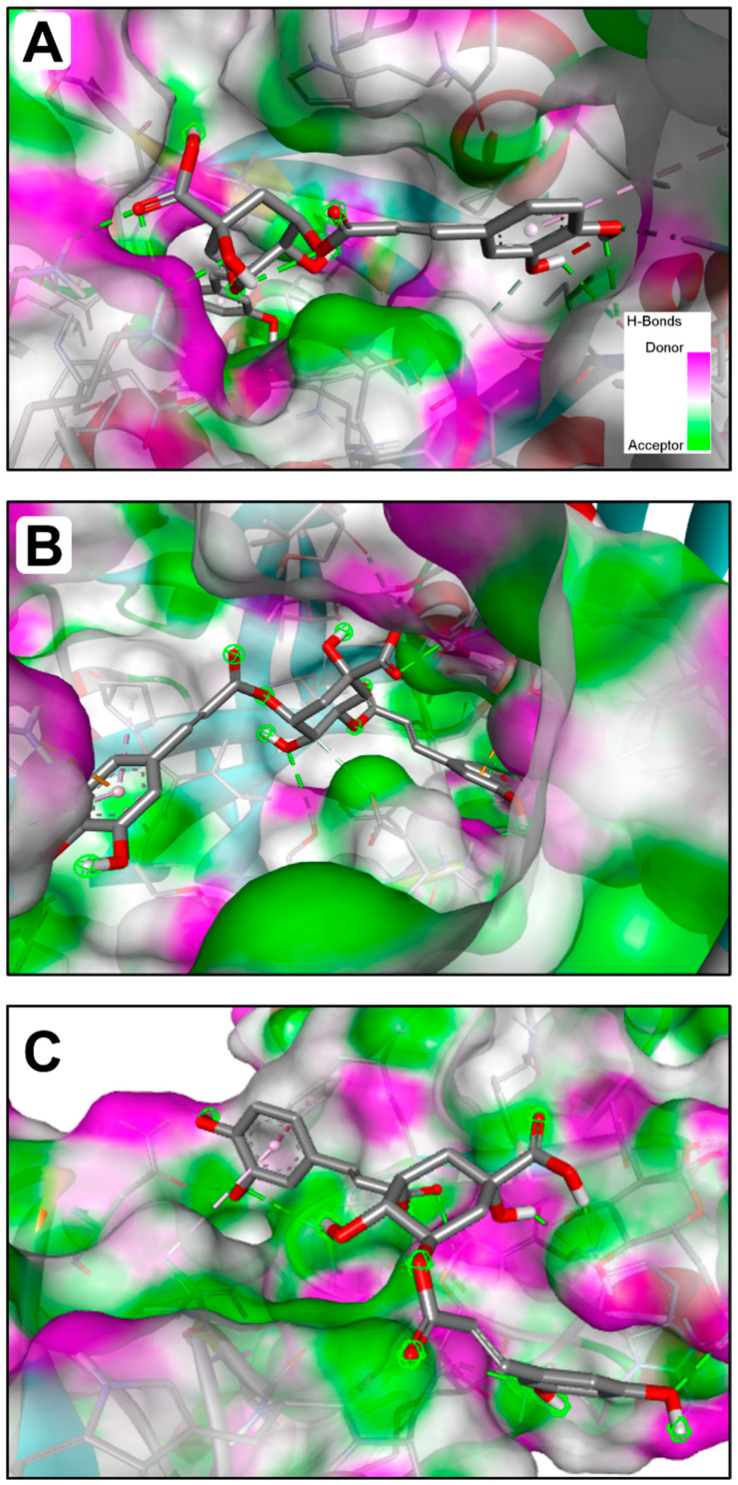
3D illustration of the H-bond interactions of (−)-3,5-dicaffeoylquinic acid (Compound No. **9**) of *P. incisa* that had the best binding affinity docket to the pocket region of the different targeted receptors: (**A**) 1JIJ, (**B**) 2XCT, and (**C**) 2UVO.

The computational analyses showed that *P. incisa* phytochemicals had various affinities to each of the targeted receptors (Table 7). Previous studies have reported that such variations are related to the chemical structures and structural geometries of ligands [53,54]. Surprisingly, all *P. incisa* phytochemicals possessed negative binding scores, which might have contributed to their biological activities. These binding affinities varied between −10.4 and −6.1 kcal/mol for 1JIJ, between −9.1 and −5.3 kcal/mol for 2XCT, and between −7.9 and −5.1 kcal/mol for 2UVO. The best binding scores were predicted for (−)-3,5-dicaffeoylquinic acid (compound **9**), followed by calamenene (compound **10**) and compounds 14, 9, or 16 for all the studied receptors, and thus the highest antimicrobial and antibiofilm activities (Table 8). (−)-3,5-Dicaffeoylquinic acid showed good molecular interactions with the targeted receptors, exhibiting 6–9 conventional H-bonds supported by a network of carbon H-bonds, hydrophobic bonds, and/or electrostatic bonds, which significantly contribute to the stability of the complexes [54,55]. These molecular interactions include several key residues: ASP40, LYS84, ARG88, VAL224, TYR36, GLN190, PRO222, ALA39, GLY193, LEU70, and VAL224 for 1JIJ; SER88, THR222, GLY34, VAL12, GLN54, ASP86, ARG51, ASP218, ARG51, and PRO120 for 2XCT; and SER43, NDG1173, NAG1174, ASN15, GLN59, PHE69, and ALA39 for 2UVO. Compound **9** was also deeply embedded in the pocket regions of 1JIJ, 2XCT, and 2UVO receptors at distances of 2.070, 1.917, and 2.176 Å, respectively (Figure 1 and Figure 2).

Overall, the calculated binding energies, molecular interactions, and deep embedding of *P. incisa* components confirm that its potential antibacterial and antibiofilm effects are thermodynamically possible. These effects have already been validated for other plants such as *Teucrium polium*, *Sargassum* sp., and *Thymus musilii* by in vitro approaches that confirmed the promising antimicrobial and antibiofilm effects of these medicinal plants and their components [53,56,57].

### 2.8. Bioavailability and Pharmacokinetics

**Figure 3:**
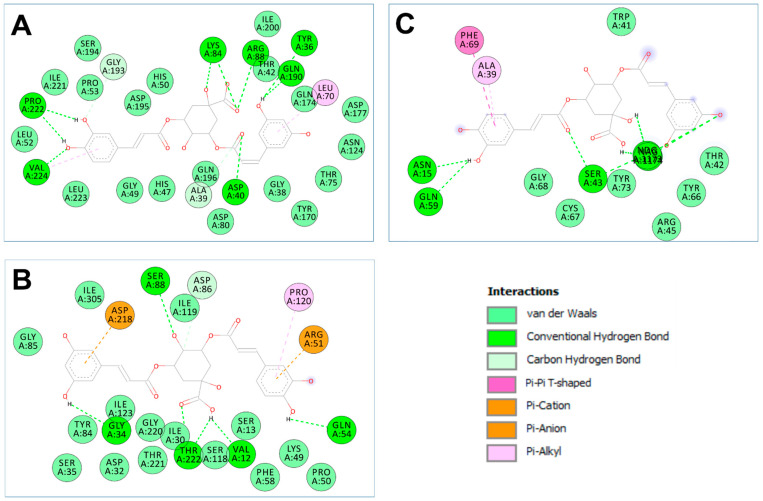
2D illustration of the diagram of interactions of (−)-3,5-dicaffeoylquinic acid (Compound No. **9**) of *P. incisa* that had the best binding affinity docket to the different targeted receptors: (**A**) 1JIJ, (**B**) 2XCT, and (**C**) 2UVO.

**Figure 4:**
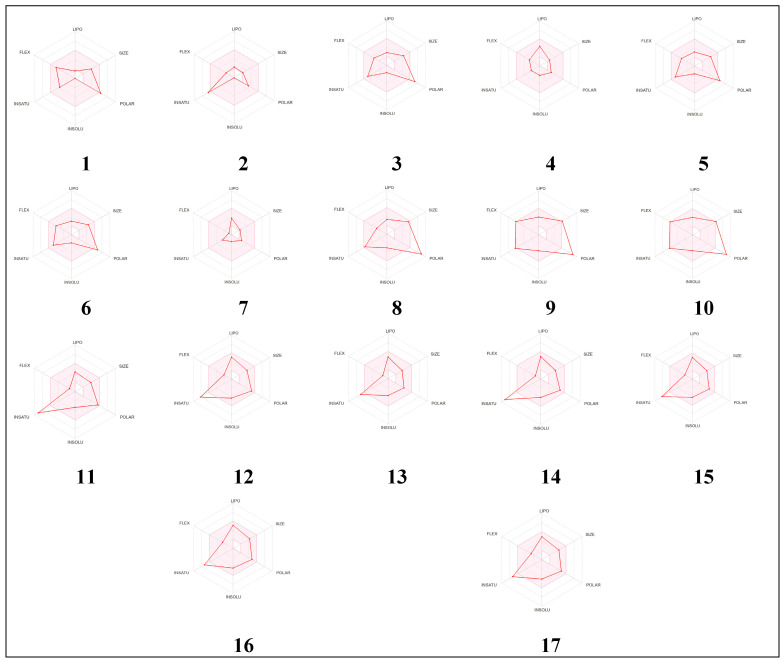
Bioavailability hexagons of *P. incisa* L. identified compounds (**1**–**17**) based on their physicochemical properties.

**Figure 5:**
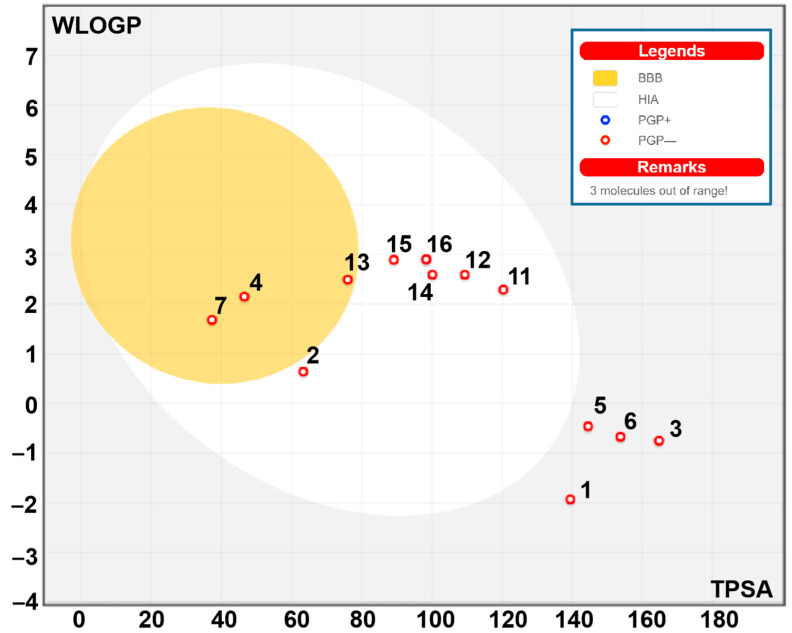
Boiled egg model of *P. incisa* L. identified compounds (**1**–**17**) based on their GI absorption, BBB permeation, and interaction with P-gp properties.

The bioavailability and pharmacokinetic properties of *P. incisa* compounds are shown in Table 9. These properties are commonly screened to avoid drug failure in the advanced stages of drug development [53,54,55]. Our results showed that except for compounds **8**, **9** and **10**, all the other compounds meet the Lipinski rule of 5. Figure 3 reports that *P. incisa* phytochemicals are suitable for oral bioavailability, as assessed by the calculation of lipophilicity (lipo), molecular size (size), polarity (pola), insolubility (insolu), unsaturation (unsatu) and flexibility (flex). These properties mediate gastrointestinal (GI) absorption and blood–brain barrier (BBB) permeation as well. GI absorption and BBB permeation were used for the mapping of the egg model (Figure 4 and Figure 5). Good oral bioavailability was largely reported to be associated with the relevant biological activity of both synthesized and natural compounds [55]. Other than compounds 9 and 10, all other compounds were predicted as not being the substrate of P-glycoprotein (P-gp), which indicated the possible safe use of *P. incisa* compounds without any toxicological outcome. As cytochromes P450 (CYPs) play important roles in the interaction, metabolism, and excretion of drugs, the inhibition of five CYP enzymes was evaluated. These CYPs were 1A2, 2C19, 2C9, 2D6, and 3A4. Our results indicated that the first 10 compounds inhibited none of the assessed CYPs, which further indicated no metabolism and excretion disruptions. Log Kp values varied between −5.86 and −10.24, indicating low to moderate skin permeation. *P. incisa* compounds are also easy to synthesize as their synthetic accessibility values ranged between 1.46 and 5.32.

## 3. Materials and Methods

### 3.1. Plant Identity and Extraction Methods

**Figure 6:**
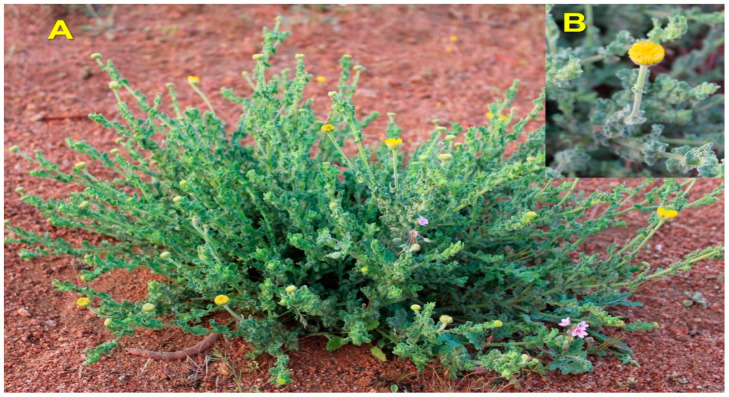
*Pulicaria incisa* collected from the Ha’il region ((**A**): Whole plant; (**B**): Flower and leaves).

*Pulicaria incisa* (Lam.) DC. (Figure 6) plant material was collected during the flowering stage from the Ha’il region of Saudi Arabia in February 2023 at the latitude 27°02′34.1″ N 42°06′53.6″ E. The species was identified by Prof. Ahmed Alghamdi, and a voucher specimen coded as PS 01 was deposited at the herbarium of the Biology Department (College of Science, University of Ha’il, Hail, Kingdom of Saudi Arabia). The plant material was air-dried at laboratory temperature (24 ± 2 °C) for several days and subsequently finely ground for further analysis. For the investigation, 40 g of fine powder was mixed with 400 mL of sterile distilled water or methanol in a 500 mL amber glass bottle and macerated for 72 h at lab temperature. Aqueous extracts were obtained after lyophilization, while the methanolic extract was obtained by removing the methanol through a rotary evaporator under vacuum. The obtained extracts were maintained in a refrigerator until use.

### 3.2. Chemical Analysis

Liquid chromatography high-resolution electrospray ionization mass spectrometry (LC-HRESIMS/MS) was used to analyze the extracts using an LTQ-XL Ion Trap mass spectrometer (Thermo Fisher Scientific, Rodano, Italy) equipped with an UltiMate 3000 HPLC (Agilent Technology, Cernusco sul Naviglio, Italy). A Kinetex Polar C18 column (100 × 3.0 mm, 100 Å, 2.6 µm) (Phenomenex, Torrance, CA, USA) was used for chromatographic separation. The injection volume was 0.5 mL/min, and the mobile phase was a mixture of A (0.1% formic acid in water, *v*/*v*) and B (0.1% formic acid in acetonitrile); a linear gradient was utilized, ranging from 5 to 60% B in 25 min, from 60 to 95% B in 10 min, and holding at 95% B for 5 min. In the positive mode, the HRMS and MSn spectra were recorded in data-dependent acquisition mode, causing fragmentation of the five strongest peaks for each scan. The ESI source conditions were the following: spray voltage of 4.8 kV; capillary voltage of 31 V; auxiliary gas of 15 (arbitrary units); sheath gas of 32; capillary temperature of 285 °C; normalized collision energy of 30; isolation width of 2.0; activation Q of 0.250; and activation duration of 30 ms. The measurement range was 150–1500 *m*/*z*.

### 3.3. Antibacterial Activity

To ensure sterility, the extracts and DMSO underwent ultrafiltration before their use in the study. The minimum inhibitory concentration (MIC) of both aqueous and methanolic extracts was determined using a modified version of the resazurin method developed by Sarker and Nahar [58]. A resazurin solution was prepared by dissolving 270 mg of resazurin in 40 mL of sterilized deionized water. In 96-well microtiter plates, the first row received 100 μL of samples in DMSO (1:10 *v*/*v*), while all other wells received 50 μL of Luria–Bertani broth or normal sterile solution. Serial dilutions of the extracts were performed in descending concentrations. To each well, 10 μL of the resazurin indicator solution was added. Furthermore, 30 μL of 3.3× sensitized broth and 10 μL of bacterial suspension (5 × 10^6^ cfu/mL) were added to each well. The plates were sealed with parafilm to prevent dehydration. A column of the plate contained the broad-spectrum antibiotic tetracycline, which was previously suspended in DMSO and served as a positive control. The negative control consisted of Luria–Bertani broth containing resazurin and bacteria without any samples. The plates were incubated at 37 °C (35 °C for *A. baumannii*) for 24 h. Visual observation was used to assess any color changes. If the solution changed from dark purple to pink or colorless, it was recorded as a positive result. The MIC value was determined as the lowest concentration of extracts that could prevent the color change from dark purple to pink.

#### 3.3.1. Antibiofilm Experiments

The inhibitory activity of the aqueous and methanolic extracts of *P. incisa* against the mature bacteria indicated above was evaluated using crystal violet and MTT tests [59].

#### 3.3.2. Crystal Violet Assay

To evaluate the inhibitory activity of the extracts on mature biofilm, flat-bottomed 96-well microtiter plates were employed [60]. Bacterial cultures were adjusted to a 0.5 McFarland standard with fresh culture broth. Each well of the microtiter plate received 10 μL of the bacterial cultures and was incubated for 24 h at 37 °C (35 °C for *A. baumannii*). After removing planktonic cells, in each well, 10 or 20 μL/mL of the extracts were added. The final volume in each well was adjusted to 250 μL with varying amounts of Luria–Bertani broth. The plates were covered with parafilm tape to prevent evaporation and incubated at 37 °C (35 °C for *A. baumannii*) for another 24 h. After removing the planktonic cells, the sessile cells were washed twice with sterile PBS. Subsequently, the plates were left under a laminar flow hood for 10 min to fix the sessile cells and then removed after 15 min. The plates were allowed to dry, and the sessile cells were stained with 200 μL of a 2% *w*/*v* crystal violet solution per well for 20 min. The staining solution was discarded, and the plates were gently washed with sterile PBS. The bound dye was released by adding 200 μL of 20% *w*/*v* glacial acetic acid. The absorbance was measured at λ = 540 nm using a Cary Varian spectrophotometer (Cary Varian, Palo Alto, CA, USA). The biofilm inhibitory activity of the extracts was calculated as the percentage relative to the control (cells grown without the samples were considered to have 0% inhibition). Triplicate tests were performed, and average results were calculated for reproducibility.

#### 3.3.3. MTT Assay

To evaluate the effect of the extracts on the metabolic activity of bacterial cells within the biofilm, the 3-(4,5-dimethylthiazol-2-yl)-2,5-diphenyltetrazolium bromide (MTT) colorimetric method was employed [60]. Two concentrations of the extracts (10 and 20 μL/mL) were added after 24 h of bacterial incubation, performed as described above, after removing the planktonic cells. After another 24 h incubation, the planktonic cells were removed, and 150 μL of PBS and 30 μL of 0.3% MTT were added. The micro plates were then incubated for two hours at 37 °C (35 °C for *A. baumannii*). The MTT solution was removed, followed by two washing steps with 200 μL of sterile physiological solution. Finally, 200 μL of dimethyl sulfoxide (DMSO) was added to suspend the formazan crystals, and the absorbance was measured at λ = 570 nm (Cary Varian, Palo Alto, CA, USA).

### 3.4. Antioxidant Activity

#### 3.4.1. DPPH Assay

The antiradical activity of the stable 1,1-diphenyl-2-picrylhydrazyl radical (DPPH) was measured using the protocol of Xiang and coworkers [61] with slight modifications. In its radical state, DPPH possesses an absorption band at 515 nm that vanishes in the presence of antiradical chemicals. To produce final concentrations ranging from 31.25 to 1000 µg/mL, aliquots of extracts were dissolved in methanol. In a straight-sided cuvette, an aliquot of methanol solution containing different concentrations of either methanolic or aqueous extracts was added to a DPPH solution (60 μM) to a final volume of 1 mL. As a control, an identical quantity of DPPH solution was applied to the cuvette, and methanol alone was used as the blank. After 45 min, the absorbance at 515 nm was measured with a Multiskan GO spectrophotometer (Thermo Fisher Scientific, Vantaa, Finland).

The result was expressed as the IC_50_ value, which is the sample concentration required to lower DPPH absorbance by 50%. The standard was 6-hydroxy 2,5,7,8-tetramethylchroman-2-carboxylic acid (Trolox, Sigma-Aldrich, Milan, Italy).

#### 3.4.2. FRAP Assay

The FRAP test was carried out in accordance with the methodology of Zhang and coworkers [62,63] with the following modifications. At an acidic pH, antioxidant chemicals are tested for their capacity to decrease the complex of Fe(III)-2,4,6—tripyridyl—s—triazine (also known as [Fe (III)—(TPTZ)2]^3+^) to Fe (II), [Fe(II)—(TPTZ)2]^2+^. The resulting complex is colored (navy blue). At a wavelength of 593 nm, the reaction may be spectrophotometrically examined. The FRAP reagent was made up of 23 mM acetate buffer (pH 3.6), 10 mM tripyridyl triazine (TPTZ) in 400 mM HCl, and 20 mM FeCl_3_. To generate the calibration curve, several quantities of ferrous sulfate heptahydrate, FeSO_4_ 7H_2_O, ranging from 27.801 mg/L to 278.010 mg/L, were made. The reaction took place in a well with a final volume of 272 µL. For 30 min, the reaction mixture was incubated in a dark environment at 37 °C. The absorbance of the FRAP alone blank was subtracted from the absorbance of the FRAP with the samples. The FeSO_4_ 7H_2_O calibration curve [64] was used to calculate the FRAP values, which were reported as the mol Fe^2+^/g of extract. The reference standard was 6-hydroxy 2,5,7,8-tetramethylchroman-2-carboxylic acid (Trolox, Sigma-Aldrich, Milan, Italy).

#### 3.4.3. ABTS Test

The ABTS test was performed using the method described by Xiang and coworkers [61]. In ultrapure water, we made a solution of ABTS 2,2′-azinobis(3-ethylbenzothiazoline-6-sulfonic acid) diammonium salt (7 mM) and ammonium persulfate (2.45 mM) (Sigma-Aldrich, Milan, Italy). To create the ABTS radical (ABTS^•^), ammonium persulfate was added to the ABTS solution until the final ammonium persulfate concentration was 2.45 mM. The sample was incubated at room temperature in the dark for 12–16 h. At 734 nm, the concentration of the ABTS radical (ABTS^•^) stock solution was determined to have an absorbance of 0.700 using a Multiskan GO spectrophotometer (Thermo Fisher Scientific, Vantaa, Finland). 6-hydroxy 2,5,7,8-tetramethylchroman-2-carboxylic acid (Trolox, Sigma-Aldrich, Milan, Italy) was utilized as an antioxidant standard. Trolox (2.5 mM) was produced and used as a stock standard in methanol. The working standards were generated and diluted with methanol on a regular basis. In triplicate, 10 μL of the standard solution or samples and 190 μL ABTS^•^ were added to the wells for analysis. Amounts of 10 μL of PBS and 190 μL of ultrapure water were added to the wells for the control (0 mM Trolox). The results are presented as milligrams of Trolox equivalent (TE) per gram of extract.

### 3.5. Analysis of Total Phenolic and Flavonoid Compounds

The total phenolic content (TPC) of the extracts was determined by spectrophotometry using the Folin–Ciocalteau technique [64]. In the cuvettes, 10 μL of diluted extract or gallic acid standard solution, 790 μL of deionized water, and 50 μL of the Folin–Ciocalteau reagent were inserted. One hundred and fifty microliters of Na_2_CO_3_ was added after 8 min. The absorbance values of the combination were measured at 765 nm after 2 h of incubation at room temperature using a Multiskan GO spectrophotometer (Thermo Fisher Scientific, Vantaa, Finland). The standard of the calibration curve was determined using gallic acid. The total phenolic content was measured in milligrams of gallic acid equivalent (mg GAE) per gram of the extract.

The total flavonoid content (TFC) was determined spectrophotometrically using the aluminum chloride colorimetric technique, as described by Baba and Malik [65], with modifications. Fifty microliters of the diluted extract or quercetin standard solution and 30 μL of 5% NaNO_2_ were added into a cuvette and incubated for 5 min. After the incubation, 30 μL of 10% AlCl_3_ was added and kept at room temperature for 5 min, then 200 μL of NaOH (1M) was supplemented to each sample and the volume was brought up to 1 mL with water; subsequently, the samples were read using a spectrophotometer at 510 nm. To make the calibration curve, quercetin was applied as a reference and the total flavonoid concentration was represented as mg quercetin equivalent (mgQE)/g of the extract.

### 3.6. Anti-Enzymatic Activities

#### 3.6.1. Cholinesterase Inhibition

Cholinesterase inhibition was assessed using the method of Zheng and coworkers [66], with minor changes. In a total volume of 1 mL, 415 μL of Tris–HCl buffer 0.1 M (pH 8), 10 μL of the extract buffer solution (in 0.1% DMSO) at various concentrations (10, 5, 2.5, 1, and 0.5 mg/mL), and 25 μL of solution containing 0.28 U/mL of AChE (or BChE) were incubated for 15 min at 37 °C. The mixture was then incubated for 30 min at 37 °C with a 1.83 mM (75 μL) solution of AChI (or BChI) and 475 μL of DTNB (5,5′-dithiobis (2-nitrobenzoic acid)) and subsequently, the absorbance at 405 nm was read using a spectrophotometer (Thermo Fisher Scientific, Vantaa, Finland). Galantamine was used as a positive control.

#### 3.6.2. α-Amylase Inhibition Assay

Jaradat’s approach, with minor modifications, was used to assess the amylase activity [67]. One hundred microliters of the extracts at various concentrations were mixed with 200 μL of 20 mM sodium phosphate buffer (pH = 6.9) and 100 μL of amylase solution (10 U/mL). For 10 min, the mixture was incubated at 37 °C. Then, 180 μL of 1% soluble starch solution was added and incubated for 20 min at 37 °C. A total of 180 μL of DNSA (3,5-dinitrosalicylic acid) solution was added to the mixture and heated for 10 min in a block heater set to 100 °C. The samples were cooled by adding 600 μL of distilled water. A UV spectrophotometer (Thermo Fisher Scientific, Vantaa, Finland) was used to measure the absorbance of the solution at 540 nm.

#### 3.6.3. α-Glucosidase Inhibition Assay

α-Glucosidase inhibitory activity was determined as previously described [68] with minor modifications. In brief, 150 μL of 0.1 M phosphate buffer at pH 7.0 was added to each well and then 10 mg of the extracts dissolved in methanol to produce varied concentrations was added. The reaction was originated by adding 15 μL of the α-glucosidase enzyme water solution (1 U/mL) to each well in the 96-well plate and incubating at 37 °C for 5 min. Then, 75 μL of the 4-nitrophenyl-D-glucopyranoside (2.0 mM) was added and the plate was incubated for 10 min at 37 °C followed by absorbance reading at 405 nm. Acarbose and samples in phosphate buffer were used as the positive and negative controls, respectively. The results were calculated based on the equation below and the final results were presented as IC_50_ values.

% = [(A_0_ − A_1_)/A_0_] × 100

where A_0_ represents the absorbance of the control without the sample and A_1_ represents the absorbance of the sample. Sample concentration producing 50% inhibition (IC_50_) was gained by calculating the inhibition % against sample concentrations.

### 3.7. Computational Analysis and Interaction Assay

The phytochemical compounds in *P. incisa* that had been identified were used in the in silico approach to decipher their molecular interactions with some receptors linked with antimicrobial and antibiofilm effects. The 3D chemical structures of these compounds were either retrieved from the PubChem website or drawn using the ChemDraw software package Pro 12.0. The 3D crystal structure of TyrRS from *S. aureus* (1JIJ), secreted aspartic proteinase 1 from *Candida albicans (2QZW)*, and wheat germ agglutinin (2UVO) receptors were obtained from the Research Collaboratory for Structural Bioinformatics Protein Data Bank (RCSB PDB). The studied ligands and three targeted receptors were prepared, processed for minimization, and saved in a pdbqt format [55,69]. They were subjected to a CHARMM force field as previously reported after targeting the grid box by selecting some key residues within the pocket region [53,54,55].

#### Bioavailability and Pharmacokinetics

Both the bioavailability and pharmacokinetic parameters of the seventeen phytochemical compounds identified in *P. incisa* were assessed by computational approach as previously described [54,55,70]. The analytical assessment was based on the ADMET (for absorption, distribution, metabolism, excretion and toxicity) measurements [53,55,56].

### 3.8. Statistical Analysis

All experiments were conducted in triplicate. Data collected from the antioxidant and anti-enzymatic assays were analyzed using SPSS 26 statistical software with one-way ANOVA followed by Tukey’s post hoc test. The differences between individual means were considered significant at *p* < 0.05. The data on the antibacterial activities were analyzed using GraphPad Prism 6.0 software (GraphPad Software Inc., San Diego, CA, USA) with two-way ANOVA followed by Dunnett’s multiple comparisons test.

## 4. Conclusions

Overall, the current study showed that *P. incisa* contains diverse groups of phytochemicals that display substantial antibacterial activities against several Gram-positive and Gram-negative bacteria. This potency could be a result of the capability of the bioactive compounds to target specific macromolecules within bacterial cells or external parts, such as cell walls and membranes. This investigation also demonstrated that *P. incisa* has great antibiofilm and antioxidant potency. The outcomes of the molecular docking analysis indicated compounds with potential antibacterial and antibiofilm activities. Moreover, the methanolic extract was shown to be active against cholinesterases and the aqueous extract, against α-glucosidase. These results comprise the first experimental data showing that *P. incisa* extracts could be used as adjuvants in the treatment of infections, diabetes, and neurodegenerative diseases.

## Figures and Tables

**Table 1:** Composition of methanolic (PM) and aqueous (PW) extracts of *P. incisa*. Compounds were listed in order of LC-MS elution. All mass peaks are [M + H]^+^ adducts.

No.	Family	Retention Time (min)	Measured *m*/*z*	Molecular Formula	Fragment	Fragment Formula	Fragment Ion (*m*/*z*)	Δppm	Identification	PM	PW
1	Amino acid derivative	1.30	294.1545	C_12_H_23_O_7_N	[M-H_2_O + H]^+^ [M-2H_2_O + H]^+^ [M-CHO_2_ + H]^+^	C_12_H_22_O_6_N C_12_H_20_O_5_N C_11_H_22_O_5_N	276.1443 258.1342 248.1494	0.674 2.521 0.527	N-Fructosyl (iso)leucine	X	X
2	Amino acid derivative	2.10	166.0862	C_9_H_11_O_2_N	[M-NH_2_ + H]^+^ [M-CHO_2_ + H]^+^	C_9_H_9_O_2_ C_8_H_10_N	149.0594 120.0806	−1.919 −1.798	L-Phenylalanine	X	X
3	Phenolic acid	7.10	355.1019	C_16_H_18_O_9_	[M-H_2_O + H]^+^ [M-C_7_H_12_O_6_ + H]^+^ [M-C_7_H_12_O_6_-CO-H_2_O + H]^+^	C_16_H_17_O_8_ C_9_H_7_O_3_ C_8_H_5_O	337.0926 163.0391 117.0335	2.362 0.855 −0.097	Chlorogenic acid	X	X
4	α-β-Unsaturated γ-lactone	7.52	199.1328	C_11_H_18_O_3_	[M-H_2_O + H]^+^ [M-2H_2_O + H]^+^	C_11_H_17_O_2_ C_11_H_15_O	181.1221 163.1104	−1.139 −8.225	(−)-Hydroxydihydrobovolide	X	X
5	Phenolic acid	7.65	339.0926	C_16_H_18_O_8_	[M-C_7_H_12_O_6_ + H]^+^ [M-C_7_H_12_O_6_-CO + H]^+^	C_9_H_7_O_2_ C_8_ H_7_O	147.0438 119.0484	−1.469 −6.144	O-Coumaroylquinic acid	X	X
6	Phenolic acid	7.82	369.1042	C_17_H_20_O_9_	[M-C_7_H_12_O_6_ + H]^+^ [M-C_7_H_12_O_6_-CH_3_O + H]^+^	C_10_H_9_O_3_ C_9_ H_5_O_2_	177.0545 145.0285	−0.569 0.648	Feruloylquinic acid	X	
7	Unsaturated ketone	8.12	169.1222	C_10_H_16_O_2_	[M-H_2_O + H]^+^ [M-H_2_O-C_3_H_6_ + H]^+^	C_10_H_15_O C_7_H_9_O	151.1108 109.0658	−6.496 9.522	8-Hydroxycarvotanacetone	X	X
8	Flavonoid	8.30	465.1020	C_21_H_20_O_12_	[M-H_2_O + H]^+^ [M-C_6_H_12_O_6_ + H]^+^	C_21_H_19_O_11_ C_15_H_11_O_7_	447.0923 303.0497	0.363 −0.723	Isoquercetin (quercetin 3-O-β-D-glucoside)	X	
9	Phenolic acid	8.80	517.1329	C_25_H_24_O_12_	[M-H_2_O + H]^+^ [M-C_9_H_7_O_3_ + H]^+^ [M-C_9_H_7_O_3_-C_7_H_12_O_6_ + H]^+^	C_25_H_23_O_11_ C_16_H_19_O_9_ C_9_H_7_O_3_	499.1230 355.1027 163.0388	−1.058 0.849 −0.740	(−)-3,5-Dicaffeoylquinic acid	X	X
10	Sesquiterpene hydrocarbon	9.74	203.1792	C_15_H_22_	[M-CH(CH3)_2_ + H]^+^ [M-CH(CH3)_2_-CH_3_ + H]^+^	C_12_H_17_ C_11_H_15_	161.1325 147.1165	0.080 −1.883	Calamenene	X	X
11	Flavonoid	11.12	317.0652	C_16_H_12_O_7_	[M-H_2_O + H]^+^ [M-C_6_H_4_O_2_ + H]^+^ [M-C_6_H_4_O_2_-H_2_O + H]^+^	C_16_H_11_O_6_ C_10_H_9_O_5_ C_10_H_7_O_4_	299.0550 209.0448 191.0336	0.018 1.771 −1.493	Rhamnetin	X	X
12	Flavonoid	11.35	331.0806	C_17_H_14_O_7_	[M-CH_3_ + H]^+^ [M-CH_3_-H_2_O + H]^+^	C_16_H_12_O_7_ C_16_H_11_O_6_	316.0573 299.0568	−1.310 6.138	Quercetin dimethyl ether	X	X
13	Flavonoid	11.77	287.0912	C_16_H_14_O_5_	[M-H_2_O + H]^+^ [M-C_8_H_6_O + H]^+^	C_16_H_13_O_4_ C_8_H_7_O_4_	269.0801 167.0338	−2.584 −0.151	Sakuranetin	X	
14	Flavonoid	11.95	301.0705	C_16_H_12_O_6_	[M-CH_3_ + H]^+^	C_15_H_10_O_6_	286.0784	1.324	Chrysoeriol	X	X
15	Flavonoid	12.25	315.0864	C_17_H_14_O_6_	[M-CH_3_ + H]^+^	C_16_H_12_O_6_	300.0630	0.501	Dihydroxy–dimethoxyflavone	X	X
16	Flavonoid	12.32	345.0969	C_18_H_16_O_7_	[M-CH_3_ + H]^+^ [M-2CH_3_ + H]^+^	C_17_H_14_ O_7_ C_16_H_11_O_7_	330.0733 315.0509	−0.316 3.177	Dihydroxy–trimethoxyflavone	X	X
17	Benzofuran dimer	13.15	451.1380	C_25_H_22_O_8_	[M-CH_2_OH-H_2_O + H]^+^	C_24_H_18_O_6_	402.3573	−1.057	1-[9-(6-Acetyl-5-hydroxy-2-benzofuranyl)-6,7,8,9-tetrahydro-2,6-dihydroxy-6-(hydroxymethyl)-3-dibenzofuranyl] ethanone	X	

**Table 2:** Minimal inhibitory concentration (µg/mL) of aqueous and methanolic extracts against various pathogenic bacteria in comparison to reference control tetracycline.

	*A. baumannii*	*E. coli*	*L. monocytogenes*	*P. aeruginosa*	*S. aureus*
*P. incisa* (Ext. H_2_O)	35 ± 4 ^a^	35 ± 3 ^a^	35 ± 3 ^a^	33 ± 2 ^a^	36 ± 2 ^a^
*P. incisa* (Ext. MeOH)	35 ± 2 ^a^	30 ± 3 ^a^	35 ± 4 ^a^	32 ± 4 ^a^	32 ± 2 ^a^
Tetracycline	25 ± 2	25 ± 2	32 ± 1	28 ± 1	28 ± 1

Results are reported as the means ± SD of three experiments. Different letters indicate mean values significantly different at *p* < 0.05, according to a one-way ANOVA followed by Tukey’s post hoc test.

**Table 3:** Inhibitory activity of the extracts on the mature biofilm (24 h).

Percentage of Inhibition Compared to the Control				
CV (24 h)	*P. incisa* (Ext. H_2_O) 10 μg/mL	*P. incisa* (Ext. H_2_O) 20 μg/mL	*P. incisa* (Ext. MeOH) 10 μg/mL	*P. incisa* (Ext. MeOH) 20 μg/mL
*A. baumannii*	19.13 **** ± 1.02	19.37 **** ± 1.26	12.35 *** ± 0.46	19.53 **** ± 1.18
*E. coli*	4.38 * ± 0.12	25.5 **** ± 1.57	32.87 **** ± 1.88	44.62 **** ± 2.87
*L. monocytogenes*	14.93 **** ± 1.02	17.21 **** ± 1.16	3.32 * ± 0.11	18.10 **** ± 1.04
*P. aeruginosa*	24.36 **** ± 1.09	36.68 **** ± 1.44	29.32 **** ± 2.86	55.78 **** ± 2.55
*S. aureus*	8.26 ** ± 0.11	32.54 **** ± 1.43	31.31 **** ± 2.97	51.27 **** ± 2.07

The tests were performed using 10 and 20 μg/mL of extract. All tests were performed in triplicate. Results are expressed as percentages (means ± SD) and were calculated assuming the control (untreated bacteria) = zero. *: *p* < 0.1; **: *p* < 0.01; ***: *p* < 0.001; ****: *p* < 0.0001, compared with the control (ANOVA followed by Dunnett’s multiple comparisons test).

**Table 4:** Inhibitory activity of the extracts on the metabolism of the bacterial sessile cell in the mature biofilm (24 h).

Percentage of Inhibition Compared to the Control				
MTT (24 h)	*P. incisa* (Ext. H_2_O) 10 μg/mL	*P. incisa* (Ext. H_2_O) 20 μg/mL	*P. incisa* (Ext. MeOH) 10 μg/mL	*P. incisa* (Ext. MeOH) 20 μg/mL
***A. baumannii***	0.00 ± 0.00	0.00 ± 0.00	0.00 ± 0.00	0.00 ± 0.00
***E. coli***	0.00 ± 0.00	0.00 ± 0.00	0.00 ± 0.00	0.00 ± 0.00
***L. monocytogenes***	0.00 ± 0.00	0.00 ± 0.00	0.00 ± 0.00	0.00 ± 0.00
***P. aeruginosa***	0.00 ± 0.00	19.98 **** ± 0.00	0.00 ± 0.00	0.00 ± 0.00
***S. aureus***	49.77 **** ± 3.88	77.94 **** ± 4.06	12.74 **** ± 1.02	30.52 **** ± 2.84

The tests were performed using 10 and 20 μg/mL of extract. All tests were performed in triplicate. Results are expressed as inhibition percentages (means ± SD) and were calculated assuming the control (untreated bacteria) = zero. ****: *p* < 0.0001, compared with the control (ANOVA followed by Dunnett’s multiple comparisons test).

**Table 5:** Inhibitory effects of *Pulicaria incisa* extracts on AChE, BChE, and α-amylase and α-glucosidase.

	IC_50_ (µg/mL)			
	AChE	BChE	α-Amylase	α-Glucosidase
*Pulicaria incisa* (aqueous extract)	1621.5 ± 109.6 ^b^	1092.5 ± 6.4 ^b^	n.a.	393.9 ± 39.9 ^a^
*Pulicaria incisa* (methanolic extract)	292.3 ± 1.5 ^a^	236.9 ± 15.7 ^a^	n.a.	1936.5 ± 164.7 ^b^
Galantamine	0.9 ± 0.4	4.6 ± 1.5	–	–
Acarbose	–	–	11 ± 4.2	963 ± 0.8

Results are reported as the means ± SD of three experiments. Different letters indicate mean values significantly different at *p* < 0.05, according to a one-way ANOVA followed by Tukey’s post hoc test. n.a. = not active (IC_50_ > 10,000 µg/mL).

**Table 6:** Total polyphenol content (TPC), total flavonoid content (TFC), and antioxidant activity of *Pulicaria incisa* extracts.

Antioxidant Activity					
	TPC mg (GAE)/g	TFC mg (QE)/g	DPPH (IC_50_) μg/mL	FRAP mmol (Fe^2+^)/g	ABTS TEAC mg (TE)/g
*P. incisa* (ext. H_2_O)	56.6 ^a^ ± 2.1	11.24 ^a^ ± 0.8	64.27 ^b^ ± 0.58	0.93 ^a^ ± 0.09	84.42 ^a^ ± 7.2
*P. incisa* (ext. MeOH)	84.80 ^b^ ± 2.8	28.30 ^b^ ± 1.2	23.29 ^a^ ± 0.38	2.99 ^b^ ± 0.14	201.86 ^b^ ± 7.8
Trolox	–	–	3.65 ± 0.01	10.30 ^a^ ± 0.08	–

Results are reported as the means ± SD of three experiments. Different letters indicate mean values significantly different at *p* < 0.05, according to a one-way ANOVA followed by Tukey’s post hoc test.

**Table 7:** Binding affinities of identified *P. incisa* L. components (**1**–**17**) with three targeted receptors: 1JIJ, 2QZW, and 2UVO.

Compound No.	Binding Affinity (kcal × mol^−1^)		
	1JIJ	2XCT	2UVO
**1**	−8.0	−7.4	−6.8
**2**	−6.3	−6.1	−5.4
**3**	−8.6	−7.5	−7.1
**4**	−6.9	−5.7	−5.4
**5**	−8.1	−7.8	−7.0
**6**	−9.0	−7.0	−6.6
**7**	−6.1	−5.3	−5.1
**8**	−7.7	−7.8	−7.3
**9**	−10.4	−9.1	−7.9
**10**	−10.1	−8.4	−7.8
**11**	−9.6	−8.0	−6.7
**12**	−9.1	−7.8	−6.8
**13**	−8.6	−7.5	−6.8
**14**	−9.7	−8.0	−7.2
**15**	−8.4	−7.6	−6.7
**16**	−9.7	−7.8	−6.9
**17**	−7.7	−7.0	−6.8

**Table 8:** Numbers of conventional H-bonds, closest interacting residues, and distance to closest interacting residue (Å) of the major *P. incisa* L. active chemical components that had the best binding affinities with the three targeted receptors: 1JIJ, 2XCT, and 2UVO.

Receptor	Chemical Compound No.	No. Conventional H-Bonds	Closest Interacting Residues	Closest Residue (Distance, Å)	No. Closest Interacting Residues
**1JIJ**	**9**	9	Conventional H-Bond: ASP40, LYS84, LYS84, ARG88, VAL224, TYR36, GLN190, PRO222, PRO222 Carbon H-Bond: ALA39, GLY193 Hydrophobic: LEU70, VAL224	VAL224:HN (2.070)	10
	**10**	10	Conventional H-Bond: LYS84, LYS84, GLY193, GLN196, VAL224, THR75, TYR170, ASP177, ASP195, PRO220 Carbon H-Bond: GLY193, ASP195	VAL224:HN (1.980)	9
	**14**	6	Conventional H-Bond: LYS84, LYS84, LYS84, ARG88, ASP177, GLN190 Carbon H-Bond: ASP40 Electrostatic: ASP80	GLN190:OE1 (2.064)	6
	**16**	8	Conventional H-Bond: LYS84, LYS84, LYS84, ARG88, TYR36, GLN190, THR75, ASP177 Carbon H-Bond: ASP40 Electrostatic: ASP80 Hydrophobic: HIS50, LEU70	LYS84:HZ1 (2.184)	10
**2XCT**	**9**	6	Conventional H-Bond: SER88, THR222, GLY34, VAL12, THR222, GLN54 Carbon H-Bond: ASP86 Electrostatic: ARG51, ASP218 Hydrophobic: ARG51, PRO120	THR222:OG1 (1.917)	9
	**10**	6	Conventional H-Bond: ARG51, SER88, THR222, SER301, SER301, LYS49 Carbon H-Bond: GLY220 Hydrophobic: PHE58, TYR225	SER301:HG (2.023)	8
	**11**	6	Conventional H-Bond: ARG195, ASP214, SER190, ASP191, GLU193, ALA303 Carbon H-Bond: GLN227 Hydrophobic: ILE213	ASP214:HN (1.961)	8
	**14**	5	Conventional H-Bond: ARG192, THR221, ASP218, THR221, ASP37 Electrostatic: ASP218 Hydrophobic: ILE305, ALA133	ASP218:OD2 (2.340)	6
**2UVO**	**9**	10	Conventional H-Bond: SER43, SER43, NDG1173, NAG1174, NDG1173, NAG1174, NDG1173, NAG1174, ASN15, GLN59 Hydrophobic: PHE69, ALA39	SER43:HG (2.176)	7
	**10**	8	Conventional H-Bond: GLY113, NAG1176, ILE155, CYS153, GOL1177, NAG1176, ASP129, CYS153 Electrostatic: ASP129	NAG1176:H3 (2.057)	6
	**8**	10	Conventional H-Bond: NAG1176, GOL1177, GOL1177, GOL1177, NAG1176, ASP129, GOL1177, GOL1177, GOL1177, ILE155 Hydrophobic: ALA125, ALA125	NAG1176:H3 (2.092)	5
	**14**	4	Conventional H-Bond: TYR159, GOL1177, ASN101, NAG1176 Electrostatic: GOL1177, ASP129 Carbon H-Bond: GOL1177, GOL1177 Hydrophobic: ALA125, ALA125, ILE155	TYR159:HH (2.223)	7

**Table 9:** Lipophilicity, pharmacokinetics, druglikeness and medicinal chemistry of *P. incisa* L. identified compounds (**1**–**17**) based on their ADMET (for absorption, distribution, metabolism, excretion, and toxicity) properties.

Entry	1	2	3	4	5	6	7	8	9	10	11	12	13	14	15	16	17
	**Lipophilicity and Physicochemical Properties**																
**TPSA**	139.48	63.32	354.31	46.53	144.52	153.75	37.30	210.51	211.28	211.28	210.36	190.36	75.99	100.13	89.13	98.36	98.36
**Log *P*o/w (iLOGP)**	0.71	1.08	0.87	2.43	0.83	1.64	1.96	0.94	1.50	1.92	2.23	2.61	2.41	2.44	2.58	2.98	2.84
**Consensus Log *P*o/w**	−1.62	−0.01	−0.39	2.24	−0.08	−0.00	1.57	−0.48	0.83	0.92	1.63	2.20	2.25	2.18	2.47	2.52	2.43
**Log S (ESOL) solubility**	0.12	−0.08	−1.62	−2.13	−1.75	−1.84	−1.51	−3.04	−3.35	−3.36	−3.36	−4.25	−3.70	−4.06	−4.20	−4.35	−4.16
	**Pharmacokinetics**																
**GI absorption**	Low	High	Low	High	Low	Low	High	Low	Low	Low	High	High	High Yes	High	High	High	High
**BBB permeant**	No	No	No	Yes	No	No	Yes	No	No	No	No	No	Yes	No	No	No	No
**P-gp substrate**	No	No	No	No	No	No	No	No	Yes	Yes	No	No	No	No	No	No	No
**CYP1A2**	No	No	No	No	No	No	No	No	No	No	Yes	Yes	Yes	Yes	Yes	Yes	Yes
**CYP2C19**	No	No	No	No	No	No	No	No	No	No	No	No	Yes	No	No	No	No
**CYP2C9**	No	No	No	No	No	No	No	No	No	No	No	Yes	No	Yes	Yes	Yes	Yes
**CYP2D6**	No	No	No	No	No	No	No	No	No	No	Yes	Yes	No	Yes	Yes	Yes	Yes
**CYP3A4**	No	No	No	No	No	No	No	No	No	No	Yes	Yes	Yes	Yes	Yes	Yes	Yes
**Log Kp (skin permeation)**	−10.24	−8.39	−8.76	−6.01	−8.41	−8.62	−6.55	−8.88	−8.37	−8.37	−6.90	−5.99	−6.02	−5.93	−5.86	−5.97	−6.17
	**Druglikeness and Medicinal Chemistry**																
**Lipinski**	Yes	Yes	Yes	Yes	Yes	Yes	Yes	No	No	No	Yes	Yes	Yes	Yes	Yes	Yes	Yes
**Ghose**	No	Yes	No	Yes	No	No	Yes	No	No	No	Yes	Yes	Yes	Yes	Yes	Yes	Yes
**Veber**	Yes	Yes	No	Yes	No	No	Yes	No	No	No	Yes	Yes	Yes	Yes	Yes	Yes	Yes
**Ergan**	No	Yes	No	Yes	No	No	Yes	No	No	No	Yes	Yes	Yes	Yes	Yes	Yes	Yes
**Muegge**	No	No	No	No	Yes	No	No	No	No	No	Yes	Yes	Yes	Yes	Yes	Yes	Yes
**Leadlikeness**	0.55	0.55	0.11	0.55	0.56	0.55	0.55	0.17	0.11	0.11	0.55	0.55	0.55	0.55	0.55	0.55	0.55
**Synthetic accessibility**	3.96	1.46	4.16	3.43	4.07	4.27	3.19	5.32	4.84	4.81	3.30	3.40	3.11	3.06	3.22	3.31	3.52

TPSA: Topological polar surface area; GI: gastrointestinal; BBB: blood–brain barrier; P-gp: P-glycoprotein; CYP: cytochrome P450.

## Data Availability

The data presented in this study are available upon request from the corresponding author.
